# Current concepts in Alzheimer’s Disease: molecules, models and translational perspectives

**DOI:** 10.1186/1750-1326-8-33

**Published:** 2013-10-22

**Authors:** Bart PF Rutten, Harry WM Steinbusch

**Affiliations:** 1 Division of Neuroscience, Department of Psychiatry and Neuropsychology, School for Mental Health and Neuroscience (MHeNS), Faculty of Health, Medicine and Life Sciences, European Graduate School of Neuroscience (EURON), Maastricht University Medical Centre, Maastricht, The Netherlands

## Abstract

The field of neuroscience research in AD has been evolving rapidly over the last few years, and has pinpointed a number of candidate targets for molecules with crucial role in the pathophysiology of AD. Recent developments have furthermore enabled new ways of modeling the disease, while an increasing number of preclinically validated targets is currently being taken one step forward and tested in clinical trials. These recent developments are reviewed in the current Special Issues Series on “Current concepts in Alzheimer's disease research: molecules, models and translational perspectives” in a number of state-of-the-art manuscripts.

## Text

**Figure 1:**
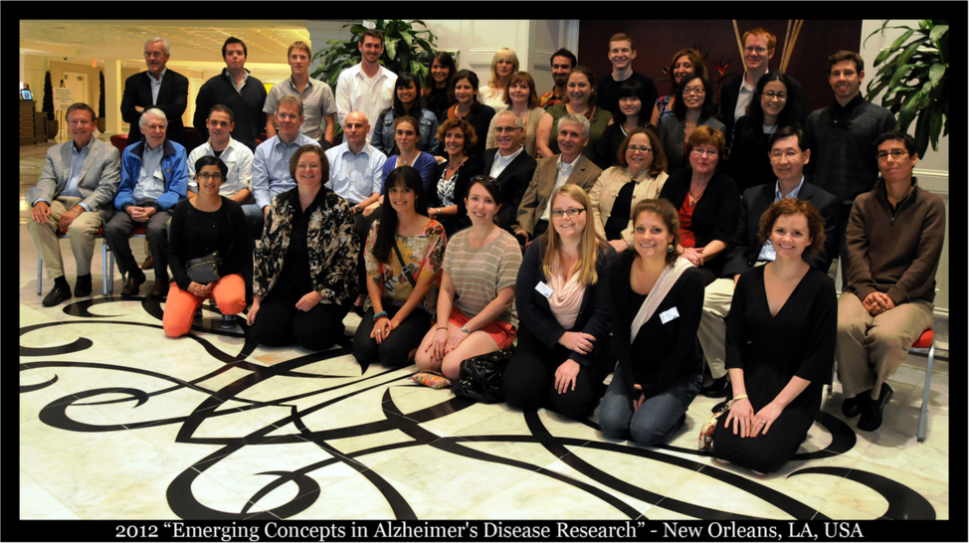
**Alzheimer’s Disease Workshop 2012: “Emerging Concepts in Alzheimer’s Disease Research” – New Orleans, Louisiana, USA.** Photo Credits: Copyright © 2012 Diane Bovenkamp, Ph.D., permission from BrightFocus Foundation. Front Row (Left to Right): Maï Panchal, Diane Bovenkamp, Paulina Davis, Rebecca Skerrett, Ingrid Heggland, Elizabeth Steuer, Gwyneth Zakaib. Second Row (Left to Right): William Klein, Paul D. Coleman, Bart P.F. Rutten, Jörg B. Schulz, Jochen Walter, Ilse Dewachter, Cynthia A. Lemere, Frank M. LaFerla, Harry Steinbusch, Stacy Haller, Carol A. Colton, Jin-Moo Lee, Joana A. Palha. Third Row (Left to Right): E. Ronald de Kloet, Edward N. Wilson, Jr., Jochen de Vry, Rylan Allemang-Grand, Julie Dela Cruz, Sarah Hescham, Romina Gentier, Julie C. Savage, Michelle Chua, Nellie Byun, Fernanda Marques, Sandro da Mesquita. Fourth Row (Left to Right): Tatiana Cerveira, Jennifer Goldman, Lionel Breuillaud, Xenos Mason, Sepideh Shokouhi and Guy Eakin. Absent: A. Claudio Cuello, Mark P. Mattson and Michael V. Sofroniew.

In October 2012, a three-day workshop on “Emerging Concepts in Alzheimer’s disease (AD)” was held in New Orleans as a Satellite Meeting to the 2012 Society for Neuroscience Meeting. The interactive workshop brought together senior and junior scientists from the entire world in a mixed format of interactive lectures and working groups of scientists assigned to jointly design innovative research projects, based on emerging concepts in AD as introduced in the lectures (Figure 1). The current Special issue Series in the journal is a direct product of the scientific exchange and discussions during the workshop. The Special Issue series comprises at least two *Molecular Neurodegeneration* editions with state-of-the-art review manuscripts by the teaching staff of the workshop, thereby covering various topics and concepts that were discussed during the workshop but also in post-workshop scientific exchanges between the participants, i.e. students and teaching staff.

### Molecules

Besides the well-known connections of the rare mutations in genes encoding for amyloid precursor protein (APP) and the presenilins (PS1 and PS2), a large body of evidence implies a crucial role for apolipoprotein E4 allele (ApoE4) in the risk of AD, e.g. recent integrative genomic analyses have described a distinct ApoE4-associated molecular pathway that promotes late-onset AD [1]. Recent findings from large genome wide association studies (GWAS) have furthermore shown evidence for associations between common variants in a set of genes, among which CLU, CR1 and PICALM, and increased risk of sporadic AD [2,3], while next generation sequencing technologies and detailed bioinformatic analyses have furthermore identified novel rare variants [4,5]. Environmental factors such as a Mediterranean diet, physical exercise, and exposure to toxins have been associated with AD, and it is likely that environmental exposures during the entire lifespan interact with genetic susceptibility in bringing about AD in the elderly [6]. Neuropathological, genetic and molecular biologic evidence has thus accumulated over the last years, and has given rise to a neurobiological theory on the cascade of events with central roles for alterations in the processing and metabolism of APP and tau protein, resulting in aggregates of beta-amyloid (Aβ) fibrils and neurofibrillary tangles. The Aβ cascade hypothesis has been fuelled with biochemical studies *in vitro* and *in vivo* studies on toxic properties of the different conformational and differently polymerized states of Aβ aggregates, particularly at the synaptic level [7,8], and has reached a more heuristic level with studies showing intricate crosstalk between misprocessing of beta-amyloid and tau proteins and neuroinflammation, ultimately disturbing neuronal and synaptic integrity and affecting cognitive functioning. A role for neuroinflammatory responses has been proposed in later phases of AD, but it has also been proposed that neuroinflammatory response act very early in the disease process by dysregulating mechanisms (for example at the level of the blood-brain barrier; [9]) to clear misfolded or damaged neuronal proteins [10,11] and heavy metals [12]. Based on recent studies indicating that dynamic changes in epigenetic regulation of gene expression is involved in many human (patho)physiological processes including experience-dependent plasticity, neurogenesis and aging, research efforts have been launched for studying epigenetic involvement in AD-associated neurodegeneration and disturbances of neuroplasticity, see e.g. [13,14]. Evidence from molecular and cellular studies have furthermore indicated that age-related changes in mitochondrial ATP production and oxidative stress are centrally involved in the pathophysiology of AD [15], while evidence reviewed by Walter et al. in the current issue suggests that membrane lipids are involved in the regulation of subcellular transport, activity, and metabolism of AD-related proteins, and that vice versa, APP and other AD-associated proteins impact on lipid metabolic pathways [16].

### Models

It is clear that no animal model will ever fully capture the complex human spectrum of molecular, cellular and functional abnormalities as seen in patients with AD, albeit that the use of animal models has been of crucial importance for breakthroughs for our understanding of the pathophysiology of AD [17]. Thus, animal models have been necessary for the identification of causal relationships of AD-related molecules, but they also offer the possibilities for *in vivo* analyses of novel intervention strategies [18]. Although transgenic mouse strains of AD are used for the majority of animal studies in AD, recent advances in the field of transgenesis have resulted into a current wave of novel rat models of aberrant APP and tau processing, which (among other advantages) enable improved behavioral phenotyping [19]. The increasing demand for large and high-throughput toxicity screens have also strengthened a position for *Drosophila melanogaster* as a useful experimental animal species, and Pruessing et al. in the current issue review the current status of Drosophila studies in relation to AD [20]. Another model system with very high potential for AD research is the use of inducible pluripotent stem cells of AD patients for neuroscience research [21,22], on which many developments are currently ongoing. Thus, modeling AD-related disturbances in neurobiological pathways using *in vivo* and *in vitro* models has undergone quite significant developments over the last few years.

### Translational perspectives

Despite the important open questions and unresolved issues in elucidating the molecular and cellular mechanisms at hand in sporadic AD cases, the AD research field is very active (however not yet successful) in bringing about therapeutic interventions that can potentially be used in clinical practice.

For example, the field of immunotherapy in AD, after finding striking effects of vaccinations in mouse models, has been one of the prime areas for translational research on therapeutic interventions over the last few years. The current status of immunotherapy (with e.g. active and passive immunization strategies) in rodent and human AD studies is reviewed by Lemere [23], who argues that (the immunological) intervention efforts may need to be targeted to individuals at risk for AD, rather than to late stage AD patients that for being effective, which off course goes hand in hand with important ethical challenges.

To summarize, AD research is expanding rapidly and is reaching the phase in which findings from fundamental neuroscience drive the development of novel diagnostic and therapeutic strategies, hopefully resulting in useful clinical tools to improve prevention and treatment of this devastating neurodegenerative disorder in the not-too-distant future.

## Abbreviations

Aβ: Beta-amyloid; AD: Alzheimer’s disease; ApoE4: Apolipoprotein E4; APP: Amyloid precursor protein; CLU: Clusterin; CR1: Complement receptor 1; PICALM: PhosphatidylInositol-binding clathrin assembly protein; GWAS: Genome wide association studies; PS1: Presenilin 1; PS2: Presenilin 2.
